# Reverse genetics construction and pathogenicity of a novel recombinant NADC30-like PRRSV isolated in China

**DOI:** 10.3389/fvets.2024.1434539

**Published:** 2024-06-26

**Authors:** Jinyao Guo, Chenxi Li, Huipeng Lu, Bin Wang, Linjie Zhang, Jingjing Ding, Xue Jiao, Qingyu Li, Shanyuan Zhu, Anping Wang, Yanhua Li

**Affiliations:** ^1^College of Veterinary Medicine, Yangzhou University, Yangzhou, Jiangsu, China; ^2^Comparative Medicine Research Institute, Yangzhou University, Yangzhou, Jiangsu, China; ^3^Jiangsu Co-Innovation Center for Prevention and Control of Important Animal Infectious Diseases and Zoonoses, Yangzhou, Jiangsu, China; ^4^Jiangsu Agri-animal Husbandry Vocational College, Jiangsu Key Laboratory for High-Tech Research and Development of Veterinary Biopharmaceuticals, Taizhou, China

**Keywords:** PRRSV, NADC30-like, RNA recombination, reverse genetics, pathogenicity

## Abstract

China has the largest pig herd in the world which accounts for more than 50% of the global pig population. Over the past three decades, the porcine reproductive and respiratory syndrome virus (PRRSV) has caused significant economic loss to the Chinese swine industry. Currently, the prevalent PRRSV strains in the field are extremely complicated, and the NADC30-like strains, NADC34-like strains, and novel recombinant viruses have become a great concern to PRRS control in China. In this study, a novel NADC30-like PRRSV, named GS2022, was isolated from the lung of a dead pig collected from a farm that experienced a PRRS outbreak. The complete genome of GS2022 shares the highest identity with the NADC30 strain and contains a discontinuous deletion of 131 aa in nsp2. Novel deletion and insertion have been identified in ORF7 and 3’UTR. Recombination analysis revealed that the GS2022 is a potential recombinant of NADC30-like and JXA1-like strains. Both inter-lineage and intra-lineage recombination events were predicted to be involved in the generation of the GS2022. An infectious cDNA clone of GS2022 was assembled to generate the isogenic GS2022 (rGS2022). The growth kinetics of rGS2022 were almost identical to those of GS2022. The pathogenicity of the GS2022 and rGS2022 was evaluated using a nursery piglet model. In the infection groups, the piglets exhibited mild clinical symptoms, including short periods of fever and respiratory diseases. Both gross lesions and histopathological lesions were observed in the lungs and lymph nodes of the infected piglets. Therefore, we reported a novel recombinant NADC30-like PRRSV strain with moderate pathogenicity in piglets. These results provide new information on the genomic characteristics and pathogenicity of the NADC30-like PRRSV in China.

## 1 Introduction

Porcine reproductive and respiratory syndrome (PRRS) is one of the most economically important infectious diseases to the pig industry worldwide. The characteristic symptoms of this disease are reproductive failure in pregnant pigs and severe respiratory syndrome in piglets. It was first reported in North America in 1987 and almost simultaneously reported in Europe, and now is widely spread globally except in Australia, New Zealand, Scandinavia, Switzerland and some South American countries.^1^ PRRSV, the etiological agent of PRRS, is a member of the genus *Betaartevirus* in the family *Arteriviridae* in the order *Nidovirales*. PRRSV can be divided into two species, *Betaarterivirus suid 1* and *Betaarterivirus suid 2*, which were also, respectively, called PRRSV-1 and PRRSV-2 (1). The prototype strains for those two species are the Lelystad and VR-2332 strains (2, 3). The single-stranded positive-sense RNA genome of PRRSV is around 15 kb in length, and the cap structure and polyadenylation tail can be found at its 5′ and 3′ ends. Two large polyproteins encoded by ORF1a and ORF1b at the 5′ end of the genome are further proteolytic processed into nonstructural proteins (nsp1α/β, nsp2 ~ nsp6, nsp7α/β, and nsp8 ~ nsp12) (4). Besides, nsp2TF and nsp2N, two additional nonstructural proteins, are translated through a dual-programmed ribosomal frameshift mechanism in the nsp2-coding region (5, 6). These nonstructural proteins play key roles in viral RNA replication and modulation of host antiviral immune responses (7, 8). At least 8 open reading frames (ORFs) at the 3′ end of the genome encode the structural proteins, including ORF2a/b, ORF3 ~ 7, and ORF5a (9).

Since the nsp2-coding region and ORF5 are the most variable regions in the PRRSV genome, their nucleotide sequences are usually used for phylogenetic analysis. A panel of unique deletion patterns has been identified in the nsp2 protein sequences of PRRSV-2, such as a discontinuous deletion of 30 amino acids (aa) in HP-PRRSV strains, a continuous deletion of 100 aa in NADC34-like strains, and a discontinuous deletion of 131 aa in NADC30-like strains (10). Based on the sequence diversity of ORF5, PRRSV-2 can be divided into nine lineages (L1 ~ L9) (11). In China, the majority of prevalent PRRSV-2 strains belong to L1, L3, L5, and L8. Since 2013, many PRRSV strains that share significantly high sequence identity with NADC30 strain reported in the US were frequently detected in south-central China and were named NADC30-like PRRSV (12–14). In 2017, two novel PRRSV strains containing a 100 aa continuous deletion in nsp2 were reported in China and termed NADC34-like PRRSV (15). In recent years, NADC30-like PRRSV and NADC34-like PRRSV have become the dominant strains circulating in pig herds in China (16). The pathogenicity of those strains varies significantly, although they are much less pathogenic to pigs than HP-PRRSV (13, 17–20). To date, the mechanism leading to the difference in pathogenicity is largely unknown.

The extremely high mutation rate and RNA recombination are the main driving forces behind the rapid evolution of PRRSV (10, 21). Recently, a large number of recombinant PRRSV strains have been reported in different regions of China, and most of the strains were generated with the NADC30-like or NADC34-like PRRSV strains as parental viruses. Consistently, PRRSV strains in lineage 1 were found to serve as parental strains in the majority of the recombination events of PRRSV-2 according to a recombination analysis of PRRSV strains from China and the US (10). However, the mechanism behind the RNA recombination and variable pathogenicity of NADC30-like PRRSV is not well understood. In this study, a novel recombinant NADC30-like PRRSV strain GS2022 was isolated from a pig lung collected from a farm experiencing PRRS outbreak in 2022 and characterized *in vitro*. An infectious cDNA clone of this strain was generated. The pathogenicity of the wild-type virus and cloned virus in piglets was further investigated.

## 2 Materials and methods

### 2.1 Cells, viruses, and antibodies

MARC-145 cells (ATCC) for PRRSV infection were cultured in Modified Eagle Medium (MEM; Sig-ma-Aldrich, St. Louis, MO, United States) supplemented 10% fetal bovine serum (FBS; Sig-ma-Aldrich, St. Louis, MO, United States) and 1% penicillin–streptomycin (Thermo Fisher Scientific, Waltham, MA, United States). BHK-21 cells (ATCC) for the recovery of recombinant PRRSV were maintained in Dulbecco’s modified eagle medium (DMEM; ThermoFisher Scientific, Waltham, MA, United States) supplemented with 10% FBS (Vazyme Biotech, Nanjing, China) and 1% penicillin–streptomycin (Thermo Fisher Scientific, Waltham, MA, United States). Porcine alveolar macrophages (PAMs) isolated from the lung lavage fluid of 4-week-age PRRSV-negative piglets were cultured in RPMI-1640 medium (Cytiva, Logan, UT, United States) containing 10% FBS (Sigma-Aldrich, St. Louis, MO, United States) and 2% penicillin–streptomycin. All cells were cultured at 37°C with 5% CO2 in a humidified incubator (ThermoFisher Scientific, Waltham, MA, United States). An HP-PRRSV TA-12 strain described previously was used in this study (22). A monoclonal antibody against PRRSV N for the immunofluorescence assay was purchased from MEDIAN Diagnostics, Korea.

### 2.2 Sample collection and virus isolation

In 2022, in a pig farm within Gansu province in China, about 60 dead piglets that had exhibited typical clinical symptoms of PRRS, including high fever, coughing, and depression, were diagnosed to be PRRSV infection by qRT-PCR (23). We further isolated a PRRSV strain with one of the PRRSV-positive lung tissues. The lung tissue was homogenized in MEM, and centrifugation was conducted to collect the virus supernatant. The supernatant was then filtrated through a 0.22 μm filter to remove bacteria contaminations. Porcine alveolar macrophages (PAMs) and MARC-145 cells were, respectively, incubated with the virus supernatant for 2 h and maintained with MEM medium supplemented with 2% FBS (Sigma-Aldrich, St. Louis, MO, United States) and 2% pen-streptomycin at 37°C. The cells were monitored daily for cytopathic effects (CPE). Virus supernatant was collected when 80% of cells exhibited obvious CPE, and cells were fixed for indirect immunofluorescence assay (IFA) with a monoclonal antibody to detect PRRSV N protein as described previously. If no CPE was observed until 5 days post-inoculation, cells were also fixed for IFA detection of PRRSV N protein. The isolated PRRSV was named as GS2022 strain and further confirmed by full-length genome sequencing.

### 2.3 Immunofluorescence assay

MARC-145 cells or PAMs were infected with the GS2022 or TA-12 viruses, respectively. At 3 dpi, cells were fixed with ice-cold methanol at −20°C for 10 min and then air-dry for immunofluorescence assay detection of PRRSV N protein. Briefly, cell monolayers were blocked with 1% BSA (Solarbio Life Sciences, Beijing, China) in 1 × PBS buffer for 30 min at room temperature, and then incubated with a monoclonal antibody against PRRSV N protein for 1 h at 37°C. After extensive washes with 1 × PBS to remove unbound primary antibody, cell monolayers were incubated with an Alexa 488-conjugated goat anti-mouse secondary antibody (Jackson ImmunoResearch Inc., West Grove, PA, United States) for 45 min at 37°C. Cell nuclei were counterstained with 4′,6-diamidino-2-phenylindole (DAPI) solution (Solarbio Life Sciences, Beijing, China). After extensive washes with 1 × PBS, fluorescent images of the cell monolayers were captured with an IX73 epifluorescence microscope (Olympus).

### 2.4 Complete genome sequencing of PRRSV GS2022 strain

To obtain the whole genomic sequence of the GS2022 strain, four pairs of primers were designed to amplify the viral genome as listed in Table 1. Viral genomic RNA was extracted from the isolated virus and the homologous of the original lung tissue using a viral nucleic extraction kit (TIANGEN Biotech, China), respectively. The cDNA generated with a HiScript III 1st Strand cDNA Synthesis Kit (Vazyme Biotech, Nanjing, China) was used for PCR amplification using the Phanta Max Super-Fidelity DNA Polymerase (Vazyme Biotech, Nanjing, China). Four DNA fragments amplified for each sample were gel-purified with a FastPure Gel DNA Extraction Mini Kit and subjected to DNA sequencing in the facility of GENEWIZ (Suzhou, China). Two consensus full-length genomes were assembled using the SnapGene software version 4.3.6 for the isolated virus and the virus contained in the lung tissue, respectively. The complete genome of GS2022 was submitted to the Genbank under Accession No. PP235415.1.

**Table 1:** A list of primers for full-length genome sequencing and reverse genetics construction.

Name	Sequence (5′-3′)	Usage
PRRSV-seq-F1	ATGACGTATAGGTGTTGG	RT-PCR amplify four overlapping genomic fragments of PRRSV
PRRSV-seq-R1	AGAAGCTCAAAAGAATGAAG	
PRRSV-seq-F2	GGTGATTGGGGYTTTGC	
PRRSV-seq-R2	TAAGGTATGTCYCCAAACCT	
PRRSV-seq-F3	ACTAAAGAGGAAGTYGCAC	
PRRSV-seq-R3	TCATTGTAATCCTCCCARTC	
PRRSV-seq-F4	AAGGAATCAGTYGCGGT	
PRRSV-seq-R4	TTTTTTTTTTTAATTACGGCCG	
GS-vec-F	GGCCGGCATGGTCCCAGCCT	PCR amplify vector
GS-vec-R	CTATTTAAATAGCTCTGCTTATATAGACCTCC	
GS-F1-F	GTCTATATAAGCAGAGCTATTTAAATATAGCATGACGTATAGGTGTTGGC	PCR amplify three overlapping genomic fragments of the GS2022
GS-F1-R	CAACCAGGTGAGTGGTTCC	
GS-F2-F	GTTTGGGAACCACTCACC	
GS-F2-R	GACCATAGACATAAGTTTGTCTCTG	
GS-F3-F	ATAAGCAGAGCTATTTAAATAGACAAACTTATGTCTATGGTCAAC	
GS-F3-R	GGAGGCTGGGACATGCCGGCCTTTTTTTTTTTTTTTTTTTTTAATTACGGCC	
GS-dnoti-F	ACGGGGAGGTCGCTGGTACCC	Inactivate the *Not*I site in the nsp1α region
GS-dnoti-R	GGGTACCAGCGACCTCCCCGTTCGTAA	

### 2.5 Sequence alignment and phylogenetic analysis

24 PRRSV genomic sequences which represent the four most prevalent PRRSV-2 lineages were downloaded for sequence alignment and phylogenetic analysis. Sequence alignment was performed with the CLC Genomic Workbench 20.0.4 (QIAGEN) for the complete genomes, nsp2-coding sequences, and ORF5 sequences of the representative PRRSV strains and GS2022 strain. Based on the aligned sequences, three phylogenetic trees were constructed, respectively, by MEGA-X using the Maximum Likelihood method and the Tamura-Nei model (24).

### 2.6 Recombinant analysis

Recombination events between PRRSV strains were detected by the Simplot software v.3.5.1 and the boot scanning analysis was performed with a window size of 400 bp and a step size of 50 bp. Furthermore, seven different algorithms (RDP, GENECONV, Bootscan, MaxChi, Chimera, SiScan, and Phylpro) included in RDP 4.0 software (25) were used to identify the potential recombination events and breakpoints in the GS2022 genome.

### 2.7 Construction of a full-length cDNA clone of the GS2022 strain

The passage 2 virus stock of GS2022 collected at 36 h post-infection (hpi) from infected PAMs was used for the construction of a full-length cDNA clone. Two rounds of homologous recombination were conducted to assemble this cDNA clone as described previously (22) with minor modifications. Viral RNA was extracted with a TIANamp Virus RNA kit (TIANGEN, Beijing, China) per the manufacturer’s instructions. Viral RNA was used as a template to synthesize cDNA using a HiScript III 1st Strand cDNA Synthesis Kit (Vazyme Biotech, Nanjing, China) using primers GS-F3-R and GS-F2-R, respectively. Three fragments (F1 ~ F3) covering the complete genome of GS2022 were PCR amplified using the Phanta Max Super-Fidelity DNA Polymerase (Vazyme Biotech, Nanjing, China), while the backbone vector was amplified using pCMV-TA-12 (22) as a template. To facilitate *in vitro* homologous recombination, the neighboring individual DNA fragments share around 20 nucleotide acids at both terminuses. Initially, the F3 fragment was inserted into the vector through homologous recombination using the NEBuilder HiFi DNA Assembly Master Mix (NEB, Ipswich, MA, United States), and the shuttle plasmid was designated as pCMV-GS2022-F3. Next, the remaining two fragments were assembled with the pCMV-GS2022-F3 which was linearized with the *Swa*I restriction enzyme using the NEBuilder HiFi DNA Assembly Master Mix (NEB, Ipswich, MA, United States). To distinguish the rescued virus from the wild-type (WT) virus, a genetic marker (two silent mutations of C564A and C567T) was introduced to disrupt the *Not*I restriction site within the nsp1α-coding region. A full-length cDNA clone of GS2022 verified by DNA sequencing was designated as pCMV-GS2022-M.

**Figure 5:**
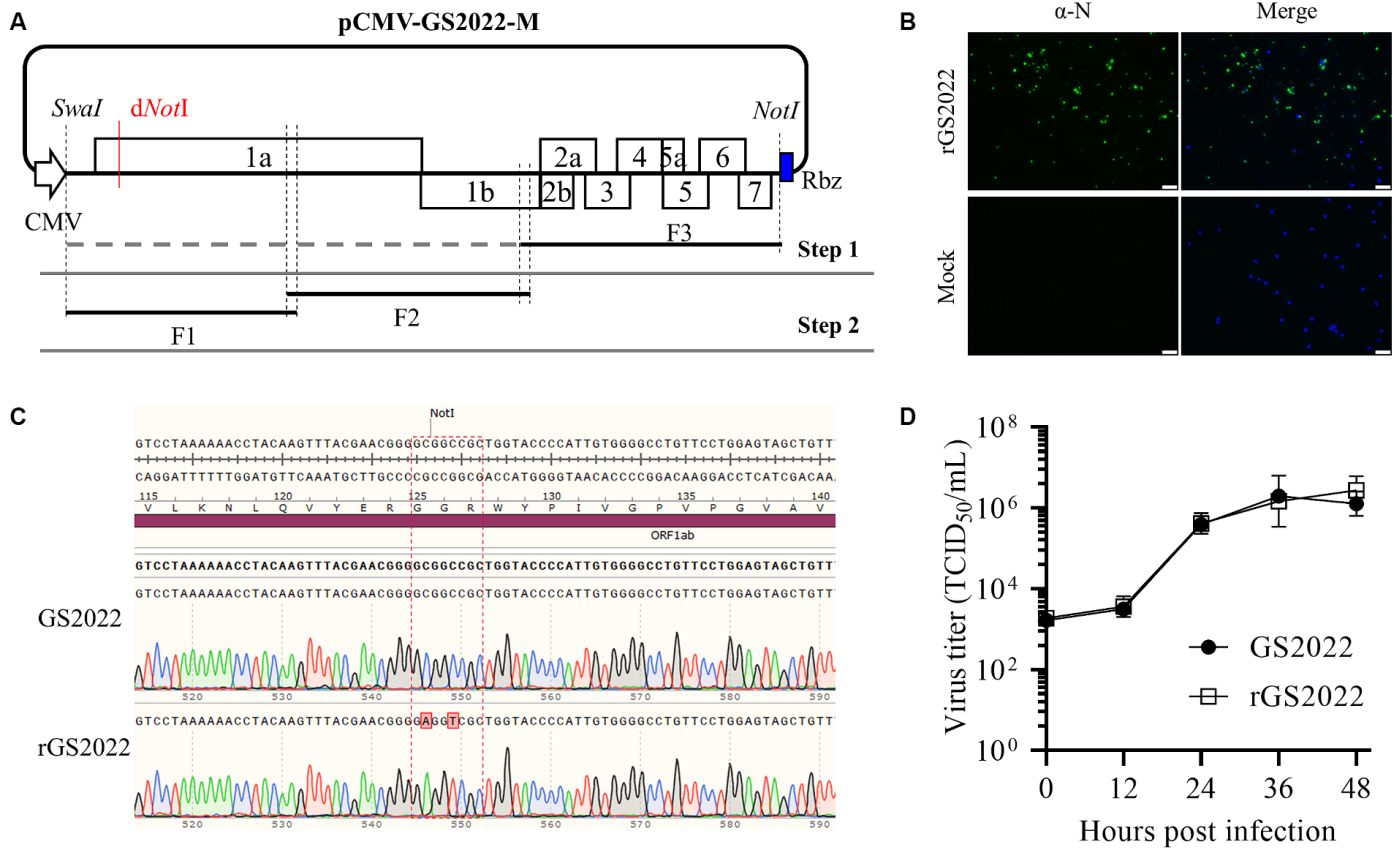
Construction of a reverse genetics of the GS2022 strain. **(A)** A schematic diagram of the construction strategy of a full-length cDNA clone. A three-step cloning strategy based on homologous recombination *in vitro* was applied to assemble a full-length cDNA clone containing a genetic marker. The complete genome of the GS2022 strain was divided into three fragments F1 ~ F3. In the first step, a human cytomegalovirus immediate-early promoter (CMV), a unique restriction enzyme site of SwaI, the F3, and a hepatitis D virus ribozyme (HDV Rbz) were inserted into the backbone vector to generate the shuttle plasmid pCMV-GS2022-F3. In the second step, the F2 and F3 were assembled with the linearized shuttle plasmid. Finally, the restriction enzyme site of *Not*I in the nsp1α-coding region was removed by two silent mutations. **(B)** Recovery and *in vitro* characterization of the recombinant viruses. BHK-21 cells in a 12-well plate were transfected with 1.5 μg cDNA clone plasmid per well using lipofectamine 3,000 transfection reagent according to the manufacturer’s instructions. At 48 hpt, culture supernatant was harvested to inoculate PAMs. When typical CPE was observed, N protein expression was confirmed by IFA detection. The nucleus was counterstained with DAPI. The scale bar is 50 μm. **(C)** The genetic marker carried by rGS2022 was confirmed by sequencing the P0 virus. **(D)** The growth curves of GS2022 and rGS2022 in PAMs. PAMs were infected with the indicated viruses at an MOI of 0.01. At the indicated time points, culture supernatants were harvested and subjected to titration in PAMs. The data points represent the means ± standard deviation (SD).

### 2.8 Recovery of the recombinant virus

DNA transfection of BHK-21 cells was performed to rescue the recombinant PRRSV. Briefly, when the BHK-21 cell monolayer in a 12-well culture plate reached about 70 ~ 80% confluence, cells were transfected with 1 μg of a full-length cDNA clone using Lipofectamine 3,000 transfection reagent (ThermoFisher Scientific, Waltham, MA, United States) according to the manufacturer’s instructions. At 2 days post-transfection (dpt), culture supernatant was harvested to infect PAMs seeded in a 12-well culture plate 12 h ahead, and BHK-21 cells were fixed with ice-cold methanol for 20 min and stained with a mAb against N protein. PAMs were monitored daily for the appearance of cytopathic effect (CPE) under an IX73 epifluorescence microscope (Olympus). Around 3 days post-infection (dpi), the culture supernatant was harvested as passage 1 (P1) virus and stored at −80°C, and then further passaged in PAMs.

### 2.9 Viral growth kinetics

The growth kinetics of the recombinant GS2022 (rGS2022) in PAMs were characterized by viral growth curves using the P3 virus. Briefly, PAMs seeded in a 24-well tissue culture plate were inoculated with GS2022 and rGS2022 at a multiplicity of infection (MOI) of 0.01, respectively. At 2 hpi, the viral inoculums were changed with fresh infection medium, MEM supplemented with 2% FBS (Sigma-Aldrich, St. Louis, MO, United States) and 2% pen-streptomycin. At the indicated time points post-infection (0, 12, 24, 36, 48 hpi), culture supernatants were harvested for viral titration and viral titers were calculated as TCID_50_/mL. For each time point, two replicates were included. Viral growth curves were plotted with viral titers using GraphPad Prism 9.

### 2.10 Animal study

All animal experiments received approval from the Institutional Animal Care and Use Committee of the Jiangsu Key Laboratory for High-Tech Research and Development of Veterinary Biopharmaceuticals, Jiangsu Agri-animal Husbandry Vocational College (protocol code jsahvc-2023-32) and strictly adhered to conventional animal welfare regulations and standards. Fifteen 4-week-old healthy piglets were purchased from a pig farm that had no previous history of PRRS outbreaks or PRRSV vaccination. These pigs were free of PRRSV, PCV2, CSFV, and PRV based on the detection of antibodies against those viruses. PRRSV-free status of these pigs was also confirmed by qRT-PCR. All pigs were randomly divided into 3 groups (*n* = 5) and housed separately in an isolated environment. Piglets in group 1 and group 2 were infected with GS2022 or rGS2022 at a dose of 10^6^ TCID_50_/mL per pig via intranasal (1 mL) and intramuscular (1 mL) routes simultaneously. The piglets of group 3 received an equivalent volume of DMEM via the same routes as the placebo. Following viral infection, the piglets underwent daily monitoring for their rectal temperature and clinical signs that were graded using a scoring system as previously reported (26), including lethargy, anorexia, skin discoloration, sneezing, coughing, labored and abdominal breathing, and respiratory rate (Supplementary Table S1). At 0, 3, 7 and 10 dpi, their body weights and blood samples were collected for further analysis. All animals were euthanized humanely at the end of the study (10 dpi). During necropsy, tissues including lung, tonsil, and submandibular lymph nodes were checked for gross lesions and collected for viral load quantification and histopathology analysis.

### 2.11 Viral RNA quantification

Viremia and viral loads in tissues were quantified by reverse-transcription quantitative PCR (RT-qPCR) assay using a primer/probe set targeting the ORF6 of PRRSV-2 (23). A standard curve generated using a serially 10-fold diluted (10^8^ to 10^3^ copies/μL) plasmid containing the ORF6 was used for absolute quantification. For the tissue samples (including lung, submandibular lymph nodes, and tonsils), approximately 0.5 g of tissue was homogenized with 1 mL of DMEM. The supernatant was collected after centrifugation at 12000× g for 10 min at 4°C to remove residual tissue debris. For each supernatant or serum sample, 140 μL was used for viral RNA extraction using the TIANamp Virus RNA kit (TIANGEN, Beijing, China), and 50 μL viral RNA was eluted. RT-qPCR reactions were assembled with 5 μL viral RNA and the HiScript II One Step qRT-PCR Probe Kit (Vazyme Biotech, Nanjing, China) according to the manufacturer’s instructions. Viral RNA loads in serum and tissues were calculated according to the standard curve.

### 2.12 Serological test

A commercial ELISA kit (NECVB, Harbin, China) was used to measure antibody response to PRRSV infection using the serum samples collected at 0, 3, 7, and 10 dpi according to the manufacturer’s instructions. The PRRSV-specific antibody titers were reported as sample-to-positive (S/P) ratios. The serum samples with an S/P ratio of 0.38 or higher were considered positive.

### 2.13 Histopathology

The tissues collected at necropsy, including the lung, lymph nodes, and tonsils were fixed in 10% buffered neutral formalin. The hematoxylin and eosin (H&E) staining were performed for pathological examination by the Servicebio (Wuhan, China).

## 3 Results

### 3.1 The isolation and characterization of the GS2022 strain

**Figure 1:**
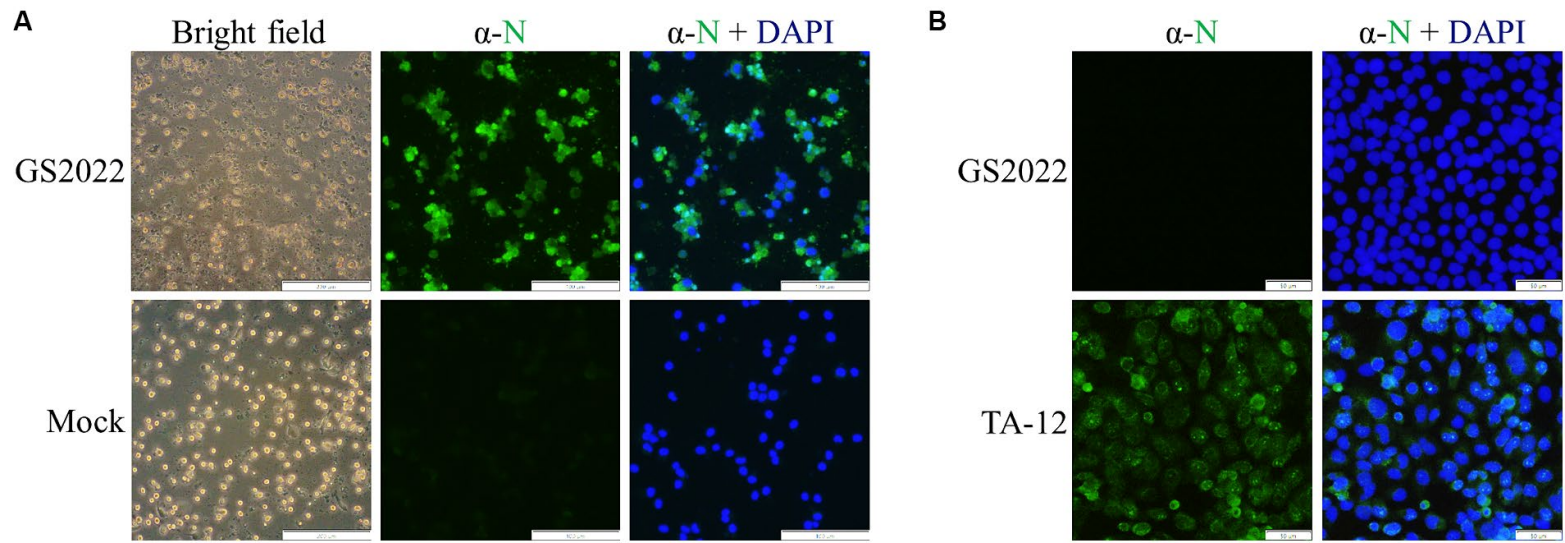
Virus isolation of GS2022 from a lung sample of a fatal piglet. **(A)** The GS2022 strain was isolated using PAMs. CPE caused by the inoculation of the lung suspension was observed at ~48 hpi. The expression of PRRSV N protein was further detected by IFA. The scale bars for bright field and fluorescent pictures are 200 μm and 100 μm. **(B)** The GS2022 virus cannot establish infection in Marc-145 cells. MARC-145 was inoculated with the isolated GS2022 and HP-PRRSV TA-12 strain, respectively. At 48 hpi, IFA detection of N protein was conducted to monitor viral replication. The scale bar is 50 μm.

A piglet lung with a high viral RNA load of PRRSV was selected for viral isolation using PAMs and MARC-145 cells. After filtration through a 0.22 μm syringe filter, the lung suspension was serially diluted for inoculation. Typical CPE characterized by irregular cell margins and cell destruction was observed at 48 hpi for PAMs with inoculation but not the mock (Figure 1A), while no CPE was observed for MARC-145 cells with inoculation until 5 dpi. This isolated PRRSV strain was designated as GS2022. To confirm the success of viral isolation, IFA was further conducted to detect the expression of viral protein using a monoclonal antibody against N protein. As expected, N protein expression in PAMs overlapped with cells that exhibited CPE (Figure 1A). In addition, we confirmed that the isolated virus was not able to infect MARC-145, while the HP-PRRSV TA-12 strain as a control can replicate in MARC-145 cells indicated by N protein expression (Figure 1B). Thus, the GS2022 strain can only infect the primary macrophages.

### 3.2 Sequence analysis of the complete genome of the GS2022 strain

**Figure 2:**
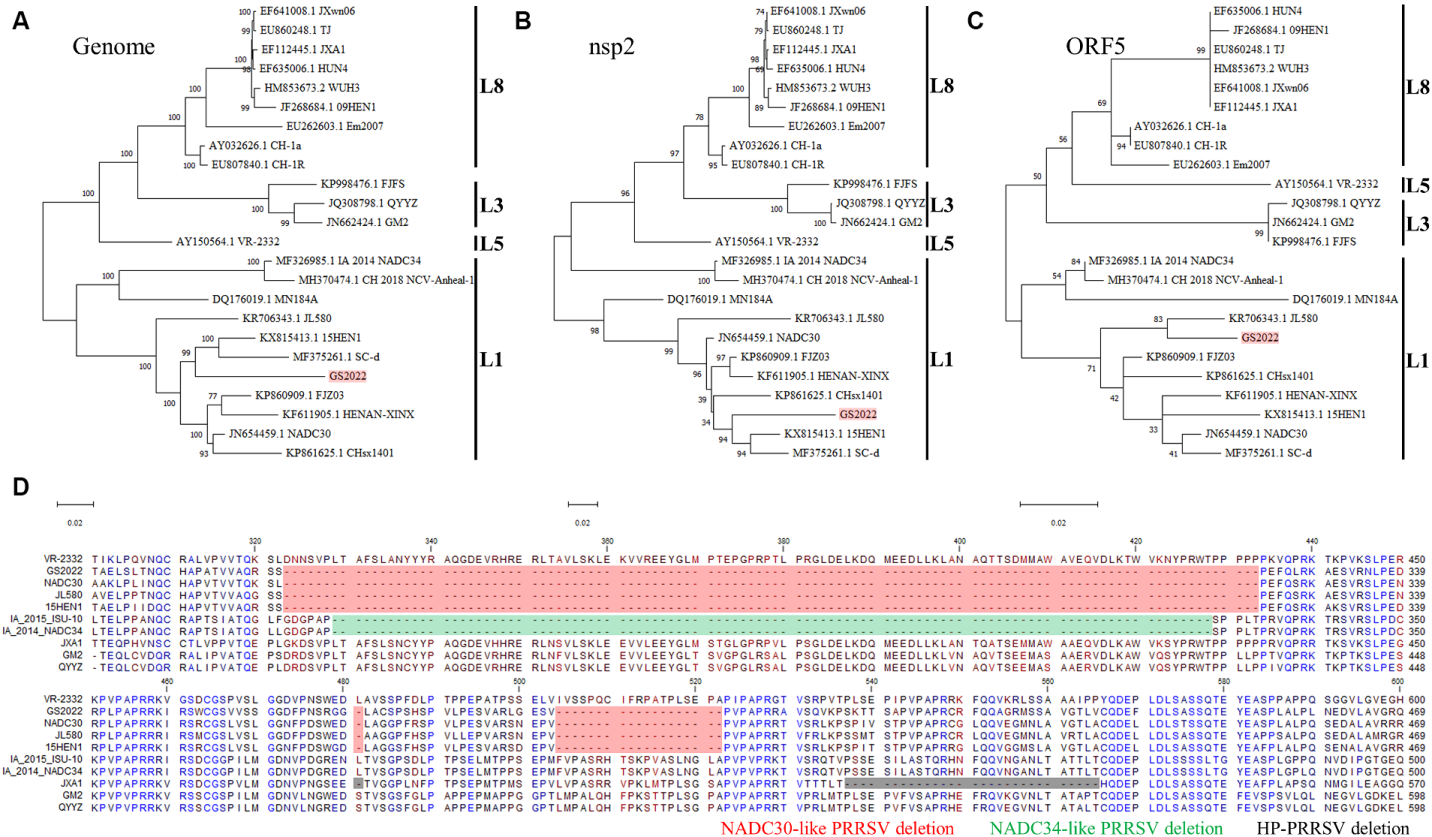
Complete genomic sequence analysis of the GS2022 strain. **(A–C)** Phylogenetic trees based on the complete genome, nsp2-coding region, and ORF5. A total of 24 complete genomes of the representative strains in lineages 1, 3, 5, and 8 were downloaded from the GenBank database for phylogenetic analysis. Sequence alignment was performed with CLC Genomic Workbench 20 (QiaGEN). Based on the aligned sequences, phylogenetic trees were constructed, respectively, by MEGA-X using the Maximum Likelihood method and the Tamura-Nei model. **(D)** Sequence analysis of nsp2 protein. The nsp2 protein of GS2022 contains a characteristic deletion pattern of 131 discontinuous amino acids at positions 323–433, 483, and 508–526. The deletion patterns specific for the NADC30-like PRRSV strain, NADC34-like PRRSV strain, and HP-PRRSV were highlighted, respectively, with colors of red, green, and black.

We downloaded all complete genomes of PRRSV-2 in the Genbank and made sequence alignment to find highly conserved regions within the PRRSV-2 genomes. Four pairs of primers (Table 1) anchoring these highly conserved regions were designed for RT-PCR amplification of the complete PRRSV genome. Four PCR products covering the full-length genome of GS2022 were purified for DNA sequencing using a panel of primers listed in Table 1. Two consensus complete genomes of GS2022 for isolated virus and lung suspension assembled with the SnapGene software were identical. Based on the BLAST-N search, GS2022 shares the highest sequence identity with two NADC30-like strains (NADC30, 91%; 15HEN1, 90.68%), but low sequence identities with the other prevalent strains in China, such as JXA1 strain (84.44%), NADC34 strain (84.08%), and VR-2332 strain (84.87%). To determine the genetic evolutionary relationship between the GS2022 strain and other representative PRRSV isolates, phylogenetic trees based on the complete PRRSV genome, nsp2-coding region, and ORF5 were constructed with 24 representative PRRSV strains that belong to the lineages of 1, 3, 5, and 8. As illustrated in Figures 2A–C, the GS2022 strain is consistently claded with the NADC30-like PRRSV strains in lineage 1. As expected, GS2022 nsp2 protein contains a characteristic deletion pattern of 131 amino acids (111 + 1 + 19) which is shared by the NADC30-like PRRSV strains (Figure 2D). Thus, the GS2022 strain is a NADC30-like PRRSV, although it shares quite low sequence identities with the other NADC30-like strains.

Next, the untranslated regions and individual coding regions of the GS2022 genome were further compared with the corresponding regions of the representative PRRSV-2 strains, respectively. As shown in Table 2, the regions of nsp1 ~ nsp2 and nsp7α ~ 3’UTR share the highest sequence identity with NADC30 strain at the levels of nucleotide and amino acid, the nsp3 ~ nsp5 is more similar to the corresponding region of the 15HEN1 strain, and the 5’UTR is more similar to that of the JXA1 strain. These results suggested that the GS2022 strain might be a recombinant virus via genomic exchange between NADC30-like PRRSV and JXA1-like PRRSV.

**Table 2:** Sequence identities between GS2022 and the representative PRRSV strains.

	NADC30		15HEN1		JXA1		NADC34		VR2332	
	nt	aa	nt	aa	nt	aa	nt	aa	nt	aa
Genome	91.00%		90.68%		84.44%		84.08%		84.87%	
5’UTR	89.42%		88.89%		**95.77%**		89.84%		90.43%	
nsp1α	**89.63%**	**93.33%**	88.52%	92.22%	89.44%	92.78%	85.37%	92.22%	88.52%	93.33%
nsp1β	**89.66%**	**83.74%**	88.51%	81.28%	79.31%	74.38%	78.16%	74.38%	80.30%	75.86%
nsp2	**89.73%**	**86.85%**	89.61%	86.01%	74.99%	69.67%	76.24%	71.80%	77.97%	72.37%
nsp3	88.26%	94.78%	**94.76%**	**98.69%**	85.88%	93.01%	85.88%	92.58%	85.44%	92.58%
nsp4	82.86%	92.16%	**95.59%**	**97.55%**	94.61%	97.06%	83.66%	92.65%	88.40%	93.14%
nsp5	88.63%	92.94%	**94.12%**	**95.29%**	92.94%	94.12%	82.16%	89.41%	87.25%	92.35%
nsp7α	**93.29%**	**93.96%**	85.01%	91.95%	84.56%	92.62%	83.67%	91.95%	87.25%	94.63%
nsp7β	**92.12%**	**92.73%**	76.97%	74.55%	78.48%	76.36%	80.00%	80.91%	83.33%	80.00%
nsp8	**94.07%**	**95.56%**	84.44%	93.33%	82.96%	91.11%	89.36%	95.56%	86.67%	91.11%
nsp9	**90.60%**	**97.08%**	88.75%	96.35%	89.73%	96.20%	86.85%	95.33%	89.00%	96.20%
nsp10	**94.63%**	**98.19%**	93.20%	97.51%	83.90%	93.65%	89.27%	97.05%	85.34%	95.24%
nsp11	**91.78%**	**95.96%**	90.88%	95.96%	87.59%	97.31%	84.60%	95.96%	87.00%	94.62%
nsp12	**94.37%**	**96.73%**	92.86%	94.12%	87.88%	94.77%	83.55%	89.54%	87.01%	92.81%
GP2a	**93.39%**	**93.75%**	93.26%	93.36%	84.44%	84.77%	85.73%	83.59%	86.64%	88.67%
GP3	**90.98%**	**90.94%**	89.15%	88.19%	81.70%	79.13%	85.10%	83.07%	83.79%	81.89%
GP4	**93.30%**	**95.51%**	90.13%	92.15%	87.50%	87.08%	91.81%	96.07%	87.34%	88.20%
GP5	**93.27%**	**89.50%**	91.54%	88.50%	84.41%	82.50%	87.89%	88.50%	84.08%	80.50%
M(6)	**95.05%**	**95.40%**	95.62%	96.55%	88.76%	94.25%	92.57%	92.53%	88.95%	93.10%
N(7)	**95.12%**	**97.54%**	93.22%	94.26%	89.10%	89.34%	93.77%	96.72%	89.70%	91.80%
3’UTR	**97.35%**		98.01%		90.67%		94.70%		91.39%	

Bold value means the highest sequence identities to the corresponding genomic regions of the GS2022 strain.

**Figure 3:**
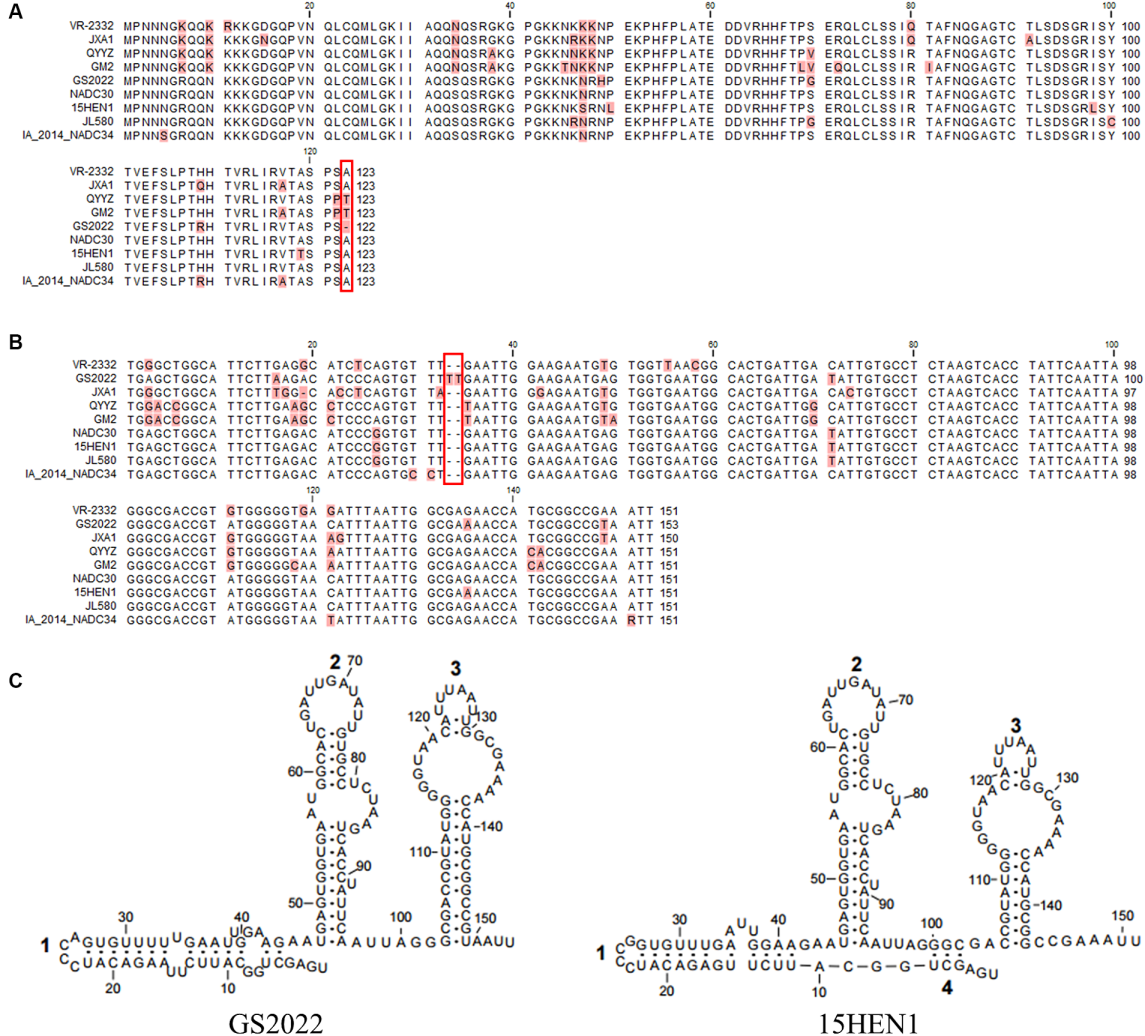
The unique deletion or insertion in ORF7 and 3’UTR of the GS2022 strain. **(A)** The N protein of GS2022 contains a deletion of the 123rd amino acid. The deletion was highlighted with a rectangle in red. **(B)** Two uridines-insertion in 3’UTR of the GS2022 strain. In the sequence alignment of 3’UTR, two uridines inserted between the 33rd and 34th positions of GS2022 3’UTR were highlighted with a rectangle in red. **(C)** The predicted RNA structures of 3’UTR. The RNA structures were predicted with the Mfold web server (27) and modified with Rnaviz 2.0.3 (28).

Besides the deletion in nsp2, a deletion of the 123rd residue of N protein was found in the GS2022 strain (Figure 3A). Through a BLAST search in the GenBank, this deletion has not been observed in any PRRSV strains reported before. Two uridines were inserted between the 33rd and 34th nucleotides in the 3’UTR of the GS2022 genome (Figure 3B). Since the secondary structure of 3’UTR may play an important role in PRRSV replication, we further compared the predicted secondary structures of the 3’UTR of the GS2022 strain and the 15HEN1 strain. Based on the prediction by MFold (Figure 3C), the structure of GS2022 3’UTR is very similar to that of 15HEN1 3’UTR, although the stem of stem-loop 1 in GS2022 is longer than that of 15HEN1.

### 3.3 GS2022 is a recombinant virus generated by inter-lineage and intra-lineage recombinations

**Figure 4:**
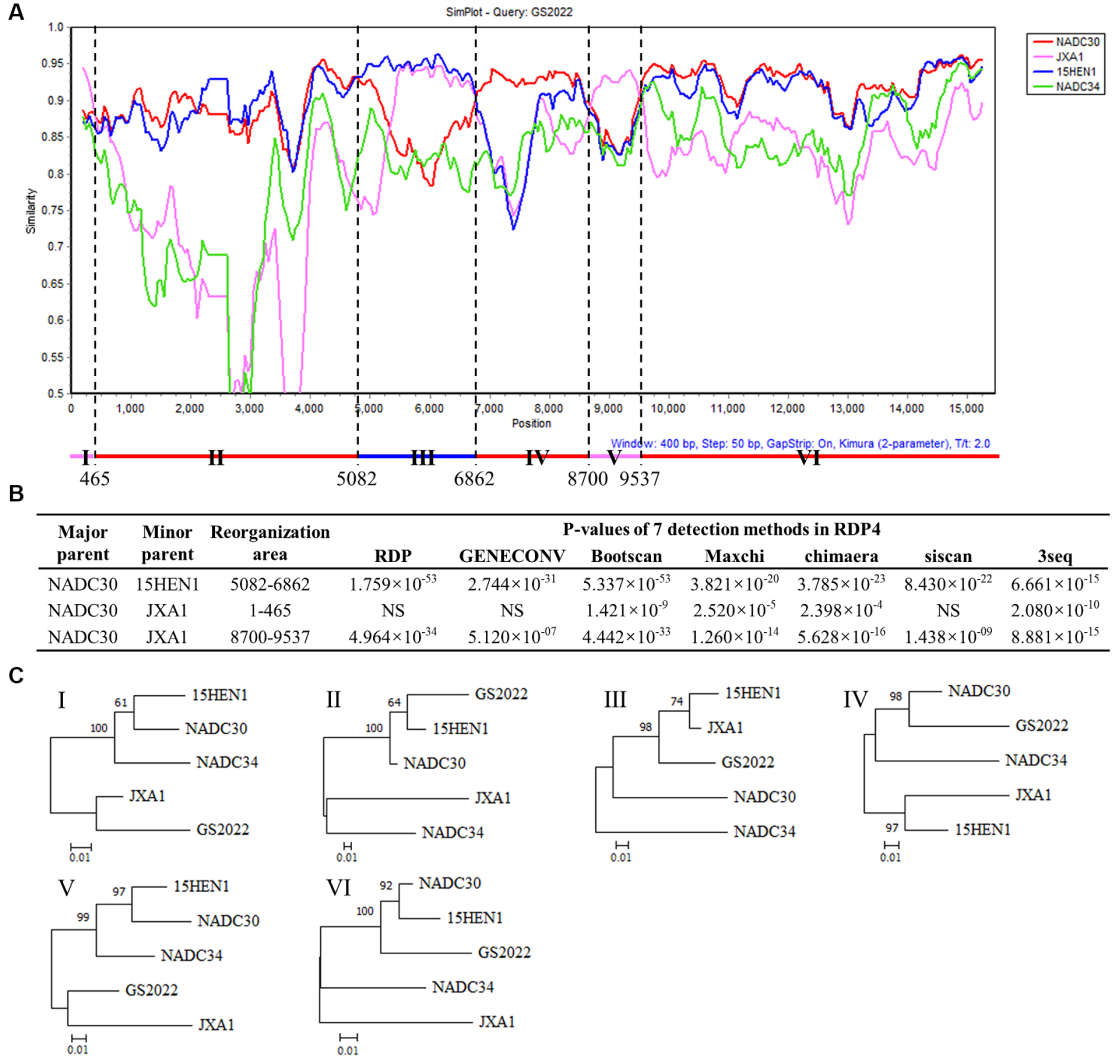
Recombination analysis of the GS2022 strain. **(A)** Recombination analysis of GS2022 was performed with Simplot v.3.5.1 software. Based on the sequence identities listed in Table 2, four representative PRRSV-2 strains were selected as the potential parental viruses for analysis, including NADC30 (JN654459), JXA1 (EF112445), 15HEN1 (KX815413.1), and NADC34 (MF326985.1). The parameters of the sliding window and step size were set as 400 bp and 50 bp, respectively. The Y-axis showed the similarity between the GS2022 and the individual representative strain. **(B)** Three recombination events of the GS2022 genome were predicted with 7 detection methods in RDP4.0. **(C)** Phylogenetic trees were constructed based on six genomic fragments divided by the predicted breakpoints.

Since the low sequence identities shared by GS2022 with the other strains in lineage 1, we speculated that GS2022 is a recombinant virus with its genomic fragments derived from multiple parental strains. Based on the sequence identities listed in Table 2, we selected four representative strains as potential parental viruses to predict recombination events of the GS2022 genome using the SimPlot software and RDP4.0. As shown in Figure 4A, the GS2022 genome is divided into six fragments by five breakpoints at nucleotide positions 465 in nsp1α, 5,082 in nsp3, 6,862 nsp7β, 8,700 nsp9, and 9,537 nsp10, which was predicted by at least 4 of 7 algorithms in RDP4.0 (Figure 4B). Based on this prediction, the GS2022 strain was generated by three crossovers among the parental viruses, NADC30 served as the major parental virus, and 15HEN1 and JXA1 served as the minor parental viruses. The predicted recombination events were further confirmed by phylogenetic analysis of six individual genomic fragments (Figure 4C). Thus, sequence analysis suggested that the GS2022 strain is a recombinant strain generated through three crossovers among NADC30, 15HEN1, and JXA1 strains.

### 3.4 Reverse genetics of the GS2022 strain

To generate a tool to study the GS2022 strain, we constructed reverse genetics of this strain through *in vitro* homologous recombination as illustrated in Figure 5A. A full-length cDNA clone of the GS2022 was verified by DNA sequencing. As shown in Supplementary Table S2, in comparison to the wild-type virus, ten mutations were identified in this cDNA clone, including 6 silent nucleotide substitutions and 4 amino acid mutations in nsp1β, nsp9, and N protein. The recombinant virus, rGS2022, was rescued by DNA transfection of BHK-21 cells with this cDNA clone and inoculation of PAMs. At 3 dpi, PAMs exhibited typical CPE, which was confirmed by IFA detection of N protein (Figure 5B). To rule out the possibility of wild-type virus contamination, the genetic marker in nsp1α of rGS2022 was confirmed by DNA sequencing (Figure 5C). The growth kinetics of rGS2022 were characterized by a viral growth curve using the P3 virus. The virus titers of rGS2022 at all time points post-infection were almost identical to those of GS2022 (Figure 5D). Thus, the reverse genetics of the GS2022 strain established here can be a useful tool for future investigation of this virus.

### 3.5 Clinical symptom and pathological lesion of GS2022-inoculated and rGS2022-inoculated piglets

**Figure 6:**
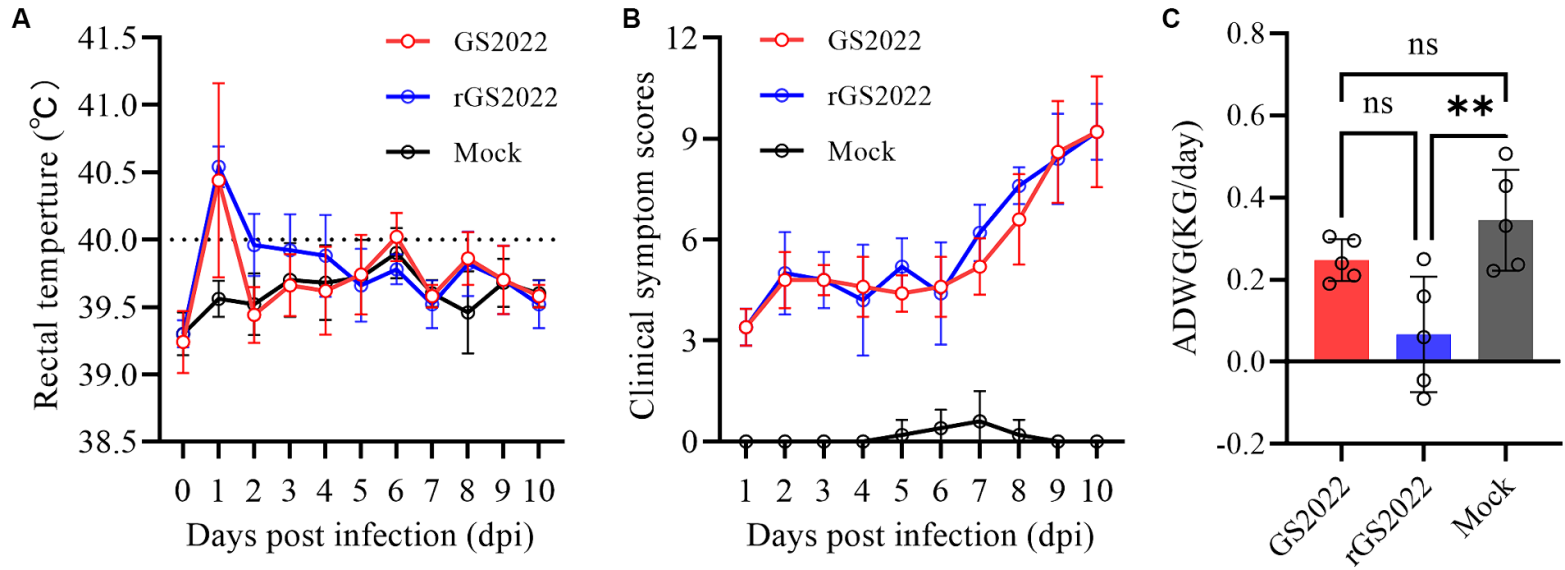
The rectal temperature, clinical sign scores, and weight gain of the piglets. **(A)** Rectal temperatures of piglets were measured daily. The clinical fever cut-off value was set at 40.0°C. **(B)** The clinical signs of each piglet were monitored daily and scored according to Supplementary Table S1. **(C)** Average daily weight gain of the piglets during the experiment (ns, *p* > 0.05, ∗∗, *p* < 0.01).

The nursery piglets were challenged with GS2022 or rGS2022 to study the pathogenicity of GS2022. A short period of fever was observed in both infection groups indicated by rectal temperatures at 1 dpi, and the rectal temperatures of pigs in the infection groups were consistently higher than those of the control group (Figure 6A). All pigs infected with GS2022 or rGS02022 showed typical clinical signs including fever, coughing, sneezing, depression, and shivering, while no obvious clinical signs were observed in the control group. The clinical signs were scored as listed in Supplementary Table S1. Since inoculation, the clinical sign scores of pigs in infection groups were gradually increasing, but not in the control group (Figure 6B). The body weight gain of the rGS2022 infection group was significantly less than that of the control group (Figure 6C). All animals survived from PRRSV challenge.

**Figure 7:**
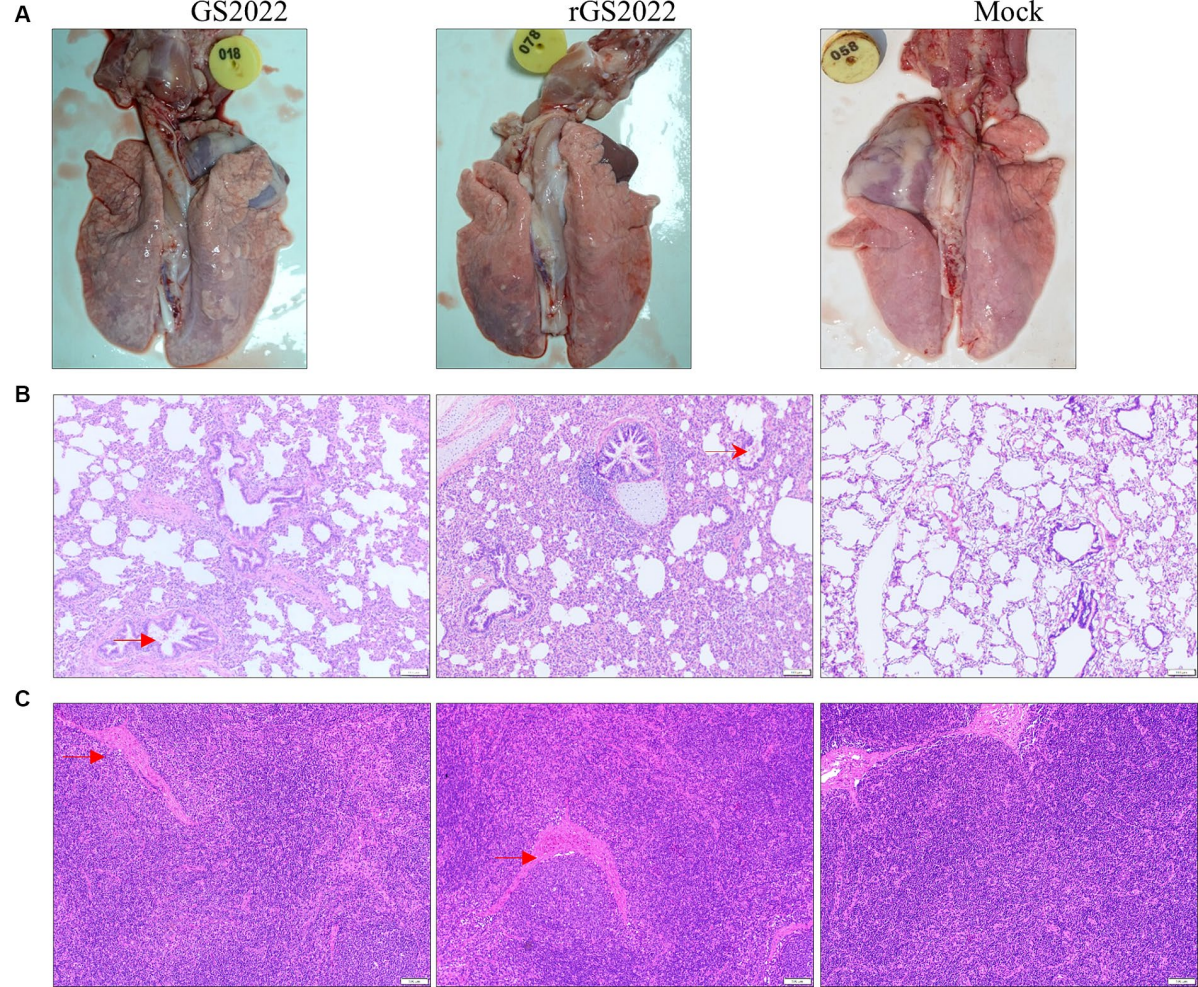
Pathological lesions in tissues. **(A)** The macroscopic lesion of the lungs. Interstitial pneumonia in the lung with thickening of alveolar septa accompanied by infiltration of immune cells hyperplasia was observed in the lungs in the PRRSV infection group. **(B)** The microscopic lesion of the lungs. H&E staining indicated the presence of interstitial pneumonia signs in the lungs of the PRRSV infection groups, such as interval thickening and inflammatory infiltration. By contrast, the alveoli of the piglets in the control group were normal. **(C)** The microscopic lesion of the submandibular lymph nodes. Vascular dilatation and infiltration of neutrophils were observed in the PRRSV infection groups. Arrows in red color were used to highlight the lesions.

In line with the results of the clinical symptoms, pigs with PRRSV infection showed different degrees of interstitial pneumonia. The lung tissues of the pigs in PRRSV infection groups exhibited moderate levels of macroscopic lesions, including pulmonary consolidation, edema, and hemorrhage (Figure 7A). Obvious microscopic lung lesions were observed in PRRSV infection groups, such as alveolar interstitial thickening and inflammatory cell infiltration (Figure 7B). Acute hemorrhage and infiltration of neutrophils were observed in submandibular lymph nodes (Figure 7C). In contrast, no pathological lesions were observed in the lung and submandibular lymph nodes of pigs in the control group (Figure 7).

### 3.6 Virema and viral load in tissues

**Figure 8:**
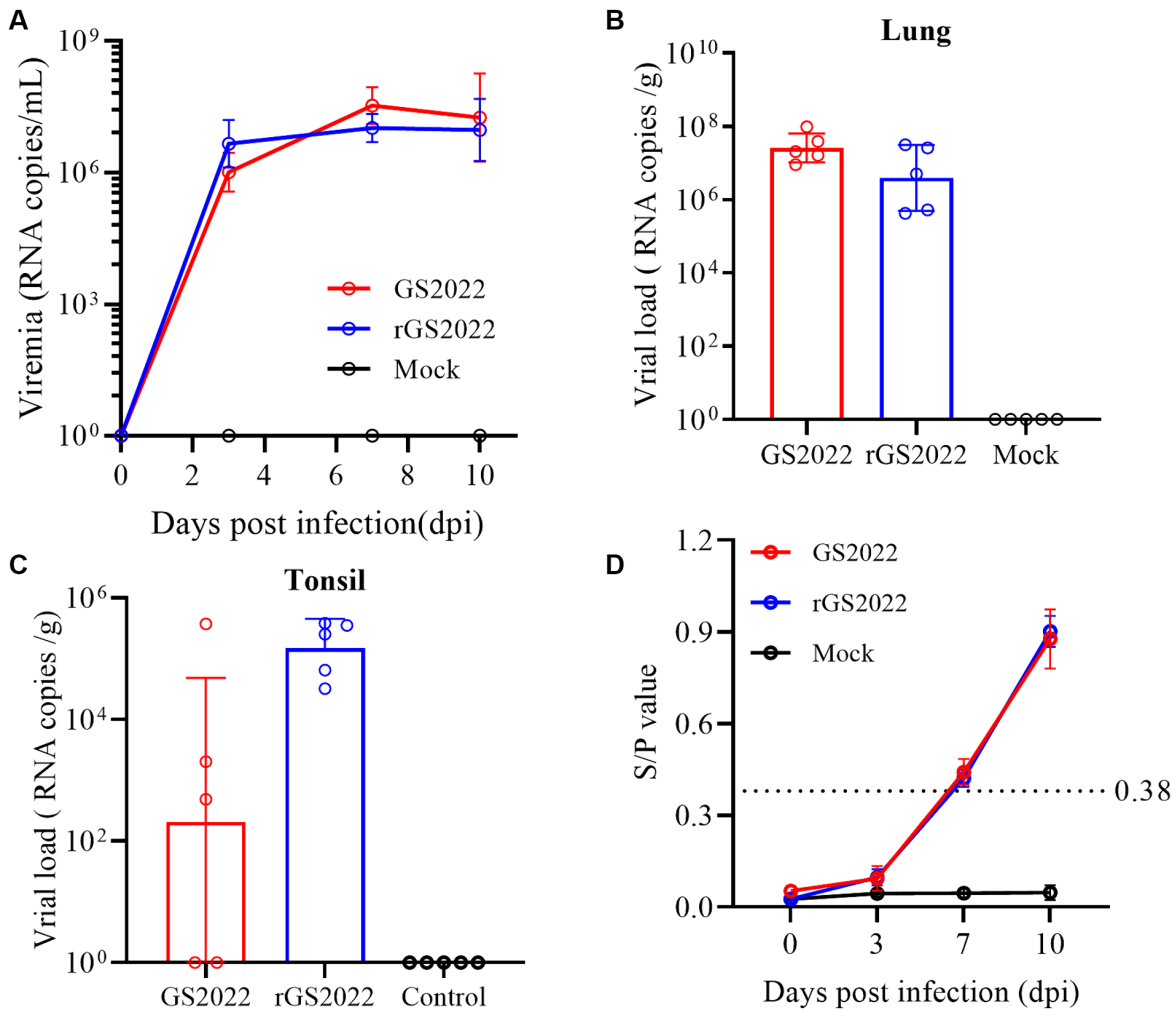
The kinetics of viremia, viral loads in tissues, and antibody responses *in vivo*. **(A)** The kinetics of viremia were evaluated by qRT-PCR. Viral RNA loads in serum samples collected at 0, 3, 7, and 10 dpi were quantified and calculated to viral genomic RNA copies/mL. **(B)** Viral loads in the lungs were determined to be viral genomic RNA copies/g. **(C)** Viral loads in the tonsils were determined to be viral genomic RNA copies/g. **(D)** PRRSV N protein-specific antibodies were measured using a commercial ELISA kit and S/*p* > 0.38 was set as the threshold of seroconversion.

To evaluate the replication of the GS2022 strain in piglets, viremia and viral load in tissues were determined by quantitative RT-PCR. The viremia of the infected pigs increased sharply from 3 dpi and peaked at 7 dpi (1 ~ 3 × 10^7^copies/mL) (Figure 8A), while no viremia was detected in the control group throughout the experimental period. Besides, viral RNA was also detected in the lung, tonsil, and lymph node tissues of pigs with infection at 10 dpi. Similar levels of viral RNA in lung tissues were detected in both infection groups (Figure 8B). In tonsil tissues, viral RNA was detected in 3/5 of pigs in the GS2022 infection group and 5/5 of pigs in the rGS2022 infection group, and higher levels of viral load were observed in the rGS2022 infection group (Figure 8C). Only 1/5 of pigs in both infection groups showed PRRSV replication in the submandibular lymph node at 10 dpi (Supplementary Table S3). Taken together, GS2022 and rGS2022 established infection in piglets and exhibited a wide distribution in tissues.

### 3.7 Humoral immune response to PRRSV infection

Antibody response to PRRSV infection was measured with a commercial ELISA kit that detected N protein-specific antibodies. In the control group, all animals remained seronegative for PRRSV-specific antibodies until the end of the experiment. In the infection groups, 4/5 pigs in the GS2022 infection group and all pigs in the rGS2022 infection group became seroconverted at 7 dpi and all pigs were seropositive at 10 dpi (Figure 8D).

## 4 Discussion

Since its first isolate reported in China in 1996, PRRSV has changed rapidly in China and led to a huge economic burden on the swine industry. In 2006, the highly pathogenic PRRSV (HP-PRRSV) outbreak in southern China that belongs to lineage 8 rapidly spread to the entire country and became the dominant strain (29). In 2013 and 2017, the NADC30-like and NADC34-like strains were, respectively, imported from the US to China and gradually replaced HP-PRRSV as the new dominant strains in the field (13–15). Since the lineage 1 strains are frequently involved in RNA recombination, a large number of recombinant PRRSV strains derived from NADC30-like and NADC34-like strains have been isolated in the field. Vaccination is one of the key strategies for PRRS control in China. A panel of PRRS vaccines derived from the classical PRRSV and HP-PRRSV strains used in the field could provide good protection against clinical diseases caused by homologous challenges but not heterologous challenges of emerging strains (30, 31). Currently, the diversified PRRSV strains in the field become a great concern to PRRS control in China. In this study, we collected clinical samples from farms experiencing PRRS-like disease outbreaks to monitor PRRSV strains emerging in the field. In the lung of a dead piglet, a PRRSV strain was isolated in PAMs but not in MARC-145 cells, and termed GS2022. Based on the complete genome analysis, GS2022 shares the highest sequence identity with NADC30, although their sequence identity is less than 91%. Phylogenetic analysis based on the complete genome, nsp2-coding region and ORF5 also supported that GS2022 is NADC30-like PRRSV-2 in lineage 1. A full-length cDNA clone of GS2022 was created to rescue the isogenic recombinant virus, rGS2022. The pathogenicity of GS2022 and rGS2022 to piglets was further determined.

Based on the estimated mutation rate of 10^−3^–10^−2^/site/year (21, 32), PRRSV has one of the highest mutation rates in RNA viruses. The accumulated mutations may lead to a shift in the pathogenicity of the mutant. For instance, critical amino acids in nsp9 determine the fatal virulence of the Chinese HP-PRRSV for piglets (33, 34). GS2022 contains a 131-aa (111 + 1 + 19) discontinuous deletion in nsp2 which was treated as a molecular marker of the NADC30-like PRRSV strains. Besides, a novel deletion of the last amino acid of the N protein was found in GS2022. This deletion has not been found in the PRRSV strains archived in the GenBank, although the last four amino acids of the N protein in PRRSV-2 were determined to be non-essential for virus infectivity (35). 3’UTR plays an important role in PRRSV replication and its secondary structure may be critical to its function (36, 37). In the 3’UTR of GS2022, the insertion of two uridines was identified between the 33rd and 34th nucleotides. Based on the prediction of Mfold, this insertion affects the structures of stem-loop 1 and stem 4, but does not change the overall structure. In the future study, we are going to further characterize the role of these mutations (deletion or insertion) in viral replication using the reverse genetics of GS2022 established in this study.

As the primary target of PRRSV, PAMs are usually collected from the lung lavage of piglets for PRRSV infection. PAMs are very costly and difficult to manipulate. Currently, the MARC-145 cell line is the most commonly used in PRRSV research, including virus isolation, vaccine preparation and investigations of PRRSV infection mechanisms. In recent years, many PRRSV-2 strains isolated using PAMs cannot establish infection in MARC-145 cells (20, 38–40). GP2a-GP3 was identified as the major determinant of PRRSV tropism in MARC-145 cells (40). The K160 residue in GP2a is associated with PRRSV infectivity in MARC-145 cells and PAMs (38). Recently, the 98th amino acid of GP2a was found to play a key role in PRRSV adaptation to MARC-145 cells, and PRRSV strains with phenylalanine at this residue fail to infect MARC-145 cells (39). In line with their finding, GS2022 carrying phenylalanine at the 98th residue of GP2a also can infect PAMs but not MARC-145 cells (Figure 1B). We will confirm whether the 98th residue of GP2a is the only determinant of PRRSV tropism in MARC-145 cells by creating mutant viruses. Due to the non-infectivity of GS2022 in MARC-145 cells, it is almost impossible to plaque purify GS2022 for reverse genetics construction. Based on the consensus sequence of GS2022, an infectious cDNA clone was assembled. Four amino acid mutations were identified between the cDNA clone and the consensus sequence of GS2022 as listed in Supplementary Table S2. The cloned virus rGS2022 exhibited very similar growth kinetics *in vitro* and *in vivo* (Figures 5D, 8A,B). In comparison to GS2022, rGS2022 infection led to slower body weight gain and higher viral load in tonsils (Figures 6C, 8C). Since rGS2022 is one of the quasispecies of GS2022, we speculate that rGS2022 and GS2022 may have some difference in pathogenicity. However, due to the limited number of animals included in each group and no significant difference in clinical symptoms of piglets between the two infection groups, it is difficult to demonstrate pathogenicity differences between GS2022 and rGS2022 with our results.

RNA recombination greatly enhanced the adaptation of PRRSV-2 strains in lineage 1 in the field (16). In recent years, many recombinant viruses have been isolated and characterized in China (19, 31, 41–44). Since the recombination patterns are quite random, the mechanism of PRRSV-2 strains in lineage 1 prone to RNA recombination remains unknown. Based on the low sequence identity (<91%) shared by GS2022 with other known strains, we speculated that GS2022 is a novel recombinant PRRSV. Recombination analysis suggested that GS2022 might be generated through three recombinations, including two inter-lineage recombinations between NADC30 and JXA1, and an intra-lineage recombination between NADC30 and 15HEN1. 15HEN1 is a recombinant PRRSV with NADC30 and JXA1-R (43). In the genome of GS2022, five breakpoints were predicted in nsp1α, nsp3, nsp7β, nsp9, and nsp10. NADC30 served as the major parental virus, while JXA1 and 15HEN1 served as the minor parental viruses. The 5′ end 465 nucleotides and the genomic region encoding nsp9 C-terminus (511 ~ 685) and nsp10 N-terminus (1 ~ 104 aa) may come from HP-PRRSV JXA1. Nsp9 and nsp10 were demonstrated to be key factors associated with the high pathogenicity of HP-PRRSV and the key residues in nsp9 have been identified (26, 33, 34). Previously, several recombinant viruses derived from NADC30 PRRSV exhibited enhanced pathogenicity in piglets or sows (41, 42, 45). The farm where the GS2022 strain was isolated has experienced a PRRS outbreak, and more than 60 pigs died from respiratory diseases. Therefore, in comparison to NADC30, GS2022 may have increased pathogenicity in piglets. Not as expected, inoculation of GS2022 and rGS2022 caused mild diseases and pathological lesions in piglets, and all animals survived the challenge. However, the limitation of this study is that the animal challenge only lasted for 10 days because of the shortage of animal facilities. Since the clinical scores were still increasing at the end of the animal study (Figure 6C), more serious clinical symptoms and death may observed if the animals have been raised for a longer time. In the future study, a panel of recombinant viruses will be generated with reverse genetics to further characterize the effect of the predicted RNA recombination on the replication and pathogenicity of GS2022.

## 5 Conclusion

In conclusion, we isolated a novel recombinant NADC30-like PRRSV in a farm experiencing a PRRS outbreak, termed GS2022. Unique deletion and insertion were identified in the GS2022 genome. An infectious cDNA clone of GS2022 was established for future investigations. Both GS2022 and rGS2022 have caused clinical symptoms and pathological lesions in piglets but no death, suggesting that GS2022 has a moderate pathogenicity in piglets.

## Data availability statement

The datasets presented in this study can be found in online repositories. The names of the repository/repositories and accession number(s) can be found at: https://www.ncbi.nlm.nih.gov/genbank/, PP235415.1.

## Ethics statement

The animal study was approved by the Institutional Animal Care and Use Committee of the Jiangsu Key Laboratory for High-Tech Research and Development of Veterinary Biopharmaceuticals, Jiangsu Agri-animal Husbandry Vocational College. The study was conducted in accordance with the local legislation and institutional requirements.

## Author contributions

JG: Formal analysis, Investigation, Methodology, Writing – original draft, Writing – review & editing. CL: Investigation, Validation, Writing – review & editing. HL: Formal analysis, Investigation, Methodology, Writing – review & editing. BW: Formal analysis, Investigation, Writing – review & editing. LZ: Formal analysis, Investigation, Writing – review & editing. JD: Investigation, Validation, Writing – review & editing. XJ: Investigation, Writing – review & editing. QL: Investigation, Writing – review & editing. SZ: Funding acquisition, Resources, Supervision, Writing – review & editing. AW: Project administration, Supervision, Writing – review & editing, Investigation. YL: Conceptualization, Formal analysis, Funding acquisition, Investigation, Methodology, Project administration, Resources, Software, Supervision, Validation, Visualization, Writing – original draft, Writing – review & editing, Data curation.

## Footnotes

^1^ https://prrscontrol.com/
