# Supplementary material for: Reverse genetics construction and pathogenicity of a novel recombinant NADC30-like PRRSV isolated in China
Source: Front Vet Sci. 2024 Jun 26;11:1434539. doi: 10.3389/fvets.2024.1434539 (PMC11237873; doi:10.3389/fvets.2024.1434539)
Supplement: Supplementary file 1 [file Table_1.docx]

**Table S1.** The scoring system of clinical signs.

|  | Clinical Symptoms | Criteria for assessment | Score |
| --- | --- | --- | --- |
| General Clinical Symptoms（GCS） | Body temperature | T≤39.9℃ | 0 |
|  |  | 40.0℃≤T≤40.9℃ | 1 |
|  |  | 41.0℃≤T | 2 |
|  | Appetite | Normal | 0 |
|  |  | Anorexia | 1 |
|  | Mental state | Normal | 0 |
|  |  | Depressed | 1 |
|  | Skin | Normal | 0 |
|  |  | Red | 1 |
| Respiratory Clinical Symptom（RCS） | Breath | Normal | 0 |
|  |  | Tachypnea (Nervously) | 1 |
|  |  | Tachypnea (Steady) | 2 |
|  |  | Tachypnea and Dyspnea | 3 |
|  |  | Tachypnea, Dyspnea, and Irregular breathing | 4 |
|  | Cough | Normal | 0 |
|  |  | Cough | 1 |
|  | Rhinorrhoea | Normal | 0 |
|  |  | Rhinorrhoea | 1 |
| Nervous system Symptoms（NSS） | Neurological symptoms | Normal | 0 |
|  |  | Shiver | 1 |
|  |  | Ataxia | 2 |
|  |  | Incongruity | 3 |
|  |  | Paralysis | 4 |
