# Supplementary material for: Reverse genetics construction and pathogenicity of a novel recombinant NADC30-like PRRSV isolated in China
Source: Front Vet Sci. 2024 Jun 26;11:1434539. doi: 10.3389/fvets.2024.1434539 (PMC11237873; doi:10.3389/fvets.2024.1434539)
Supplement: Supplementary file 2 [file Table_2.docx]

| **Position in genomic RNA** |  | **Codon** | | **Amino acid substitution** |
| --- | --- | --- | --- | --- |
|  |  | **GS2022** | **cDNA clone** |  |
| 888 | nsp1β | CA*C^a^* | CA*T* | / |
| 1122 | nsp1β | *A*TT | *G*TT | I→V |
| 1132 | nsp1β | *A*TC | *C*TC | I→L |
| 2831 | nsp2 | AG*C* | AG*T* | / |
| 3306 | nsp2 | GC*T* | GC*A* | / |
| 6822 | nsp7a | CT*T* | CT*C* | / |
| 7726 | nsp9 | A*A*A | A*G*A | K→R |
| 12189 | ORF2a | CA*C* | CA*T* | / |
| 12602 | ORF3 | AA*C* | AA*T* | / |
| 14821 | ORF7 | C*G*C | C*A*C | R→H |

**Table S2.** The mutations found in the cDNA clone of GS2022.
