# Supplementary material for: Reverse genetics construction and pathogenicity of a novel recombinant NADC30-like PRRSV isolated in China
Source: Front Vet Sci. 2024 Jun 26;11:1434539. doi: 10.3389/fvets.2024.1434539 (PMC11237873; doi:10.3389/fvets.2024.1434539)
Supplement: Supplementary file 3 [file Table_3.docx]

**Table S3.** Viral loads in submandibular lymph nodes of the inoculated piglets at 10 dpi.

| Group | Pig No. | Genome copies/g |
| --- | --- | --- |
| GS2022 | A1 | 0 |
|  | A2 | 0 |
|  | A3 | 10^4.33^ |
|  | A4 | 0 |
|  | A5 | 0 |
| rGS2022 | B1 | 0 |
|  | B2 | 0 |
|  | B3 | 0 |
|  | B4 | 0 |
|  | B5 | 10^6.42^ |
